# Hounsfield unit attenuation value can differentiate pyonephrosis from hydronephrosis and predict septic complications in patients with obstructive uropathy

**DOI:** 10.1038/s41598-020-75672-8

**Published:** 2020-10-29

**Authors:** Luca Boeri, Irene Fulgheri, Franco Palmisano, Elena Lievore, Vito Lorusso, Francesco Ripa, Mario D’Amico, Matteo Giulio Spinelli, Andrea Salonia, Gianpaolo Carrafiello, Emanuele Montanari

**Affiliations:** 1 Department of Urology, Foundation IRCCS Ca’ Granda – Ospedale Maggiore Policlinico, University of Milan, Via della Commenda 15, 20122 Milan, Italy; 2 Department of Pharmacy, Foundation IRCCS Ca’ Granda – Ospedale Maggiore Policlinico, Milan, Italy; 3 Department of Radiology, Foundation IRCCS Ca’ Granda – Ospedale Maggiore Policlinico, University of Milan, Milan, Italy; 4 Division of Experimental Oncology/Unit of Urology, URI,, IRCCS Ospedale San Raffaele, Milan, Italy

**Keywords:** Risk factors, Urology

## Abstract

We aimed to assess the role of computerized tomography attenuation values (Hounsfield unit—HU) for differentiating pyonephrosis from hydronephrosis and for predicting postoperative infectious complications in patients with obstructive uropathy. We analysed data from 122 patients who underwent nephrostomy tube or ureteral catheter placement for obstructive uropathy. A radiologist drew the region of interest for quantitative measurement of the HU values in the hydronephrotic region of the affected kidney. Descriptive statistics and logistic regression models tested the predictive value of HU determination in differentiating pyonephrosis from hydronephrosis and in predicting postoperative sepsis. A HU cut-off value of 6.3 could diagnose the presence of pyonephrosis with 71.6% sensitivity and 71.5% specificity (AUC 0.76; 95%CI: 0.66–0.85). At multivariable logistic regression analysis HU ≥ 6.3 (*p* ≤ 0.001) was independently associated with pyonephrosis. Patients who developed sepsis had higher HU values (*p* ≤ 0.001) than those without sepsis. A HU cut-off value of 7.3 could diagnose the presence of sepsis with 76.5% sensitivity and 74.3% specificity (AUC 0.79; 95%CI: 0.71–0.90). At multivariable logistic regression analysis, HU ≥ 7.3 (*p* ≤ 0.001) was independently associated with sepsis, after accounting for clinical and laboratory parameters. Measuring HU values of the fluid of the dilated collecting system may be useful to differentiate pyonephrosis from hydronephrosis and to predict septic complications in patients with obstructive uropathy.

## Introduction

The term pyonephrosis (PYO) refers to infected hydronephrosis associated with suppurative destruction of the kidney parenchyma with loss of renal function^1^. PYO is considered a urological emergency and it can rapidly progress to sepsis and septic shock^1^. Several studies have shown that the combination of obstructive uropathy and infection is the underlying cause of up to 85% of urosepsis and shock cases^2^, with a disease-related mortality rate of approximately 50%^3,4^. Therefore, rapid diagnosis and treatment of PYO are essential to avoid permanent loss of renal function and to prevent sepsis.

In clinical practice the distinction between PYO and uninfected hydronephrosis (HYDRO) is challenging and the molecular mechanisms underlying the shift from HYDRO to PYO in the presence of urinary obstruction are poorly understood^1,5^. Previous studies have described various risk factors for PYO in patients with urinary stones such as long-term disease, severe HYDRO, stone size and non-functioning kidney^5^. Moreover, measurement of computerized tomography attenuation values (Hounsfield Unit—HU) of the fluid in the dilated renal collecting system was found to be a useful marker for differentiating PYO from HYDRO in patients with obstructive uropathy^6^.

HU measurement is routinely performed in clinical practice to define hardness^7,8^ and composition^9,10^ of kidney stones, to predict the outcome of stone treatment^11,12^ and to differentiate malignant from benign renal tumors^13,14^. Computerized tomography (CT) attenuation values of bladder and renal pelvis urine have also been used to predict the positivity of urine cultures with high sensitivity and specificity^15,16^. However, very little is known about the role of HU of dilated collecting systems in predicting infectious complications in patients with obstructive hydronephrosis.

Thus, we performed a cross-sectional, real-life, observational study aimed at evaluating: (1) the prevalence and predictors of PYO and, (2) the potential impact of HU values in predicting postoperative infectious complications in a cohort of patients treated with nephrostomy tube or ureteral catheter placement for obstructive uropathy at a single academic centre.

## Results

Overall, median (interquartile) patients’ age and body mass index (BMI) were 58 (37–73) years and 25.5 (22.6–27.7) Kg/m^2^, respectively. Obstructive hydronephrosis was caused by urinary stones, urothelial tumours and other causes in 97 (79.5%), 18 (14.7%) and 7 (5.7%) cases, respectively. Stone characteristics are reported in Supplementary Table 1. Criteria suggestive for PYO were found in 46 (36.7%) of the 122 patients. Patients with PYO had higher Charlson Comorbidity Index (CCI) scores (*p* = 0.04), were more frequently of female gender (*p* = 0.04) and had a more severe hydronephrosis (*p* = 0.03) than HYDRO individuals (Table 1). Moreover, PYO patients reported higher peaks of body temperature, white blood cells count (WBC) and C-reactive protein (CRP) levels (all *p* ≤ 0.001) than those with HYDRO. Groups were similar in terms of age, BMI and serum creatinine levels. Urine cultures were positive in 40 (86.9%) patients with PYO and in 11 (14.4%) patients with HYDRO, despite the clean appearance of drained urine (Supplementary Table 2 reports urine culture details).

**Table 1:** Demographic characteristics and descriptive statistics of the study cohort according to the presence of hydronephrosis or pyonephrosis (No. = 122).

	Hydronephrosis	Pyonephrosis	*p* value*
No. of patients [No. (%)]	76 (62.3)	46 (37.7)	
Age (years)			0.1
Median (IQR)	58.0 (36–68)	58.5 (49–80)	
Range	25–81	25–89	
Gender [No. (%)]			0.04
Male	47 (61.8)	20 (43.5)	
Female	29 (38.2)	26 (56.5)	
BMI (kg/m^2^)			0.9
Median (IQR)	25.2 (22.7–27.9)	25.8 (22.0–28.0)	
Range	19.5–35.5	17.9–36.7	
CCI (value)			0.04
Median (IQR)	1.0 (0.0–3)	2.0 (0.0–3.25)	
Mean (SD)	1.6 (2.0)	2.5 (2.6)	
Range	0–8	0–11	
CCI ≥ 1 [No. (%)]	40 (52.6)	31 (67.4)	0.1
Aetiology of hydronephrosis [No. (%)]			0.5
Stones	62 (81.6)	35 (76.1)	
Urothelial tumours	11 (14.5)	7 (15.2)	
Other	3 (3.9)	4 (8.7)	
Degree of hydronephrosis [No. (%)]			0.03
II	44 (57.9)	18 (39.1)	
III–IV	32 (42.1)	28 (60.9)	
Preoperative antibiotic use [No. (%)]	26 (34.2)	18 (39.1)	0.4
Max body temperature (Celsius degree)			≤ 0.001
Median (IQR)	37.0 (36–38)	38.0 (37.2–38.6)	
Range	36.0–42.0	36.0–40.0	
White blood cells count (× 10^3^/mmc)			≤ 0.001
Median (IQR)	10.9 (8.1–13.2)	14.5 (10.5–18.8)	
Range	1.1–24.5	3.3–53.0	
C-reactive protein (mg/dL)			≤ 0.001
Median (IQR)	3.5 (0.6–9.0)	13.0 (5.1–25.5)	
Range	0.3–34.5	0.2–52.0	
Serum creatinine (mg/dL)			0.1
Median (IQR)	1.3 (1.0–1.9)	1.7 (1.3–2.2)	
Range	0.6–9.6	0.2–8.3	
Perirenal fat stranding [No. (%)]	60 (78.9)	33 (71.7)	0.4
ROI area (mm^2^)			0.3
Median (IQR)	187.5 (124.7–259.5)	182.1 (142.2–333.2)	
Range	61.4–783.5	66.2–1451.4	
ROI perimeter (mm)			0.7
Median (IQR)	51.6 (43.8–61.2)	50.5 (43.5–71.2)	
Range	28.6–99.3	29.3–135.1	
Hounsfield unit value			≤ 0.001
Median (IQR)	2.5 (− 1.1 to 7.8)	9.4 (4.9–15.8)	
Range	− 7.2 to 20.9	− 2.6 to 69.5	
Variability of Hounsfield unit			0.4
Median (IQR)	13.8 (12.0–15.4)	14.2 (12.5–16.5)	
Range	8.2–24.8	8.5–28.3	
SIRS [No. (%)]	24 (31.6)	36 (78.3)	≤ 0.001
SEPSI [No. (%)]	10 (13.1)	24 (52.2)	≤ 0.001

BMI, body mass index; CCI, Charlson Comorbidity Index; ROI, region of interest; SIRS, systemic inflammatory response syndrome.

*p value according to the Mann–Whitney test for continuous data and the Chi Square Test for categorical variables, as indicated.

We found high interobserver agreement among Radiologists for measurements of the region of interest (ROI) area (Intraclass Correlation Coefficient—ICC 0.72), ROI perimeter (ICC 0.76) and HU values of hydronephrotic region (ICC 0.9) (Table 2).

**Table 2:** Intraclass correlation coefficient test for interobserver agreement.

	Radiologist 1	Radiologist 2	*p* value*	ICC (95% CI)
ROI area (mm^2^)			0.2	0.72 (0.43–0.82)
Median (IQR)	184.3 (128.1–262.4)	179.6 (104.0–237.6)		
ROI perimeter (mm)			0.6	0.76 (0.46–0.92)
Median (IQR)	50.8 (43.1–61.4)	49.6 (36.7–64.2)		
Hounsfield unit value			0.8	0.90 (0.85–0.93)
Median (IQR)	4.6 (0.7–10.4)	4.5 (0.1–8.4)		

ICC, intraclass correlation coefficient; ROI, region of interest.

*p value according to the Wilcoxon Signed Rank Test.

Patients with PYO had higher median HU values (9.4 vs. 2.5; *p* ≤ 0.001) than those with HYDRO (Table 1). On the contrary, rates of perirenal fat stranding, median ROI area and perimeter were similar between groups. Of clinical interest, patients with PYO experienced higher rates of Systemic Inflammatory Response Syndrome (SIRS) (78.3% vs. 31.6%) and sepsis (52.2% vs. 13.1%) after surgery (all *p* ≤ 0.001).

**Figure 1:**
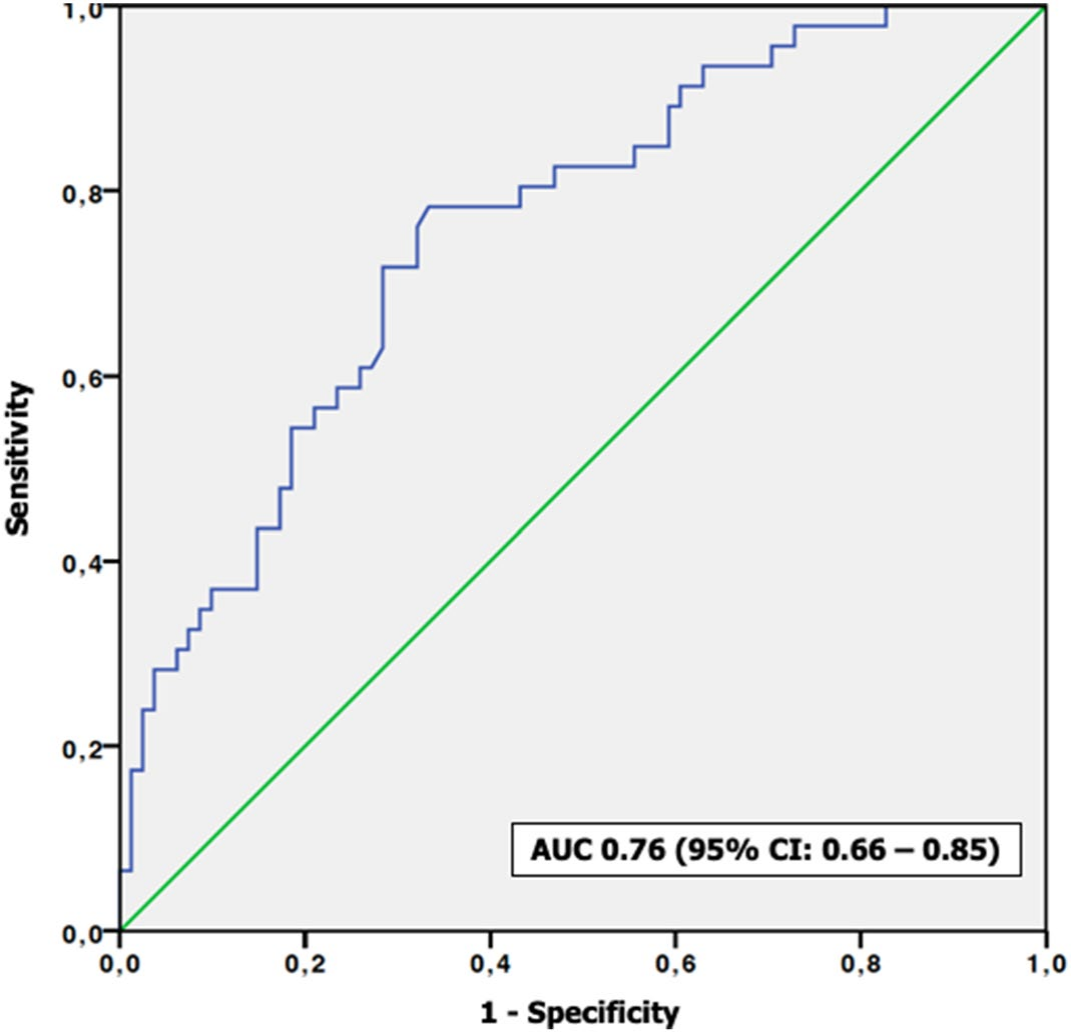
ROC analysis demonstrating the sensitivity and specificity of Hounsfield units (HU) in differentiating pyonephrosis from hydronephrosis.

Receiver Operative Characteristic (ROC) analysis revealed that HU measurements had a good ability to differentiate PYO from HYDRO (AUC 0.76; 95%CI: 0.66–0.85) (Fig. 1). In particular a HU cut-off value of 6.3 could diagnose the presence of PYO with 71.6% sensitivity and 71.5% specificity.

Table 3 depicts univariable (UVA) and multivariable (MVA) logistic regression models testing the associations between clinical variables and PYO status. At MVA, grade III-IV hydronephrosis (OR 2.73; *p* = 0.03), WBC ≥ 15 × 10^3^/mmc (OR 3.15; *p* = 0.03) and HU ≥ 6.3 (OR 8.01; *p* ≤ 0.001) were independently associated with PYO, after accounting for gender and peak body temperature.

**Table 3:** Logistic regression models predicting pyonephrosis in the whole cohort (OR; *p* value [95%CI]).

UVA model	MVA model			
	OR, *p* value	95% CI	OR, *p* value	95% CI
Age	1.03, 0.07	0.98–1.11		
BMI	1.01, 0.90	0.89–1.13		
Female gender	2.34, 0.02	1.12–4.78	1.10, 0.64	0.30–2.09
CCI ≥ 1	1.91, 0.08	0.92–3.89		
Aetiology				
Stone	Ref	Ref		
Urothelial tumours	1.31, 0.82	0.42–3.40		
Other	2.54, 0.22	0.57–4.31		
Grade III–IV	2.67, 0.01	1.14–5.17	2.73, 0.03	1.06–7.06
Vs. grade II hydronephrosis				
Perirenal fat stranding	0.62, 0.231	0.22–1.49		
Max body temperature	1.73, < 0.01	1.20–2.45	1.44, 0.07	0.96–2.17
WBC ≥ 15 × 103/mmc	4.88, ≤ 0.001	2.12–10.85	3.15, 0.03	1.08–9.11
C-reactive protein	1.13, ≤ 0.001	1.08–1.23		
Serum creatinine	1.21, 0.51	0.85–1.39		
HU ≥ 6.3	6.41, ≤ 0.001	2.86–12.32	8.01, ≤ 0.001	2.92–12.43

UVA, univariate model; MVA, multivariate model; BMI, body mass index; CCI, Charlson Comorbidity Index; WBC, white blood cells; HU, Hounsfield unit.

Since we found a positive association between PYO and the development of postoperative SIRS and sepsis, we tested the relationship between HU and sepsis.

Table 4 shows demographic characteristics of patients as segregated according to the presence of postoperative sepsis. Patients with sepsis were more frequently female (*p* < 0.01), had higher CCI (*p* = 0.02), higher max body temperature and inflammatory markers (all *p* ≤ 0.001) compared to those without sepsis. Interestingly, patients who developed sepsis had higher HU values of the hydronephrotic collecting system (12.4 vs. 2.7; *p* ≤ 0.001) than those who did not experience sepsis. Spearman’s correlation revealed that HU values were positively associated with the Sequential Organ Failure Assessment (SOFA) score (rho = 0.24, *p* ≤ 0.01).

**Table 4:** Demographic characteristics and descriptive statistics of the study cohort according to the presence of sepsis (No. = 122).

	− Sepsi	+ Sepsis	*p* value*
No. of patients [No. (%)]	88 (72.1)	34 (27.9)	
Age (years)			0.6
Median (IQR)	58.0 (41–68)	57.0 (39–79)	
Range	25–89	25–88	
Gender [No. (%)]			< 0.01
Male	56 (63.6)	11 (32.4)	
Female	32 (36.4)	23 (67.6)	
BMI (kg/m^2^)			0.9
Median (IQR)	25.3 (23.0–27.9)	25.6 (22.0–28.2)	
Range	17.9–35.5	19.5–36.7	
CCI (value)			0.02
Median (IQR)	1.0 (0.0–3.0)	2.0 (0.0–5.0)	
Mean (SD)	1.6 (2.0)	2.7 (2.5)	
Range	0–11	0–8	
CCI ≥ 1 [No. (%)]	46 (52.3)	25 (73.5)	0.03
Aetiology of hydronephrosis [No. (%)]			0.6
Stones	71 (80.7)	26 (76.5)	
Urothelial tumours	13 (14.8)	5 (14.7)	
Other	4 (4.5)	3 (8.5)	
Degree of hydronephrosis [No. (%)]			0.3
II	47 (53.4)	15 (44.1)	
III–IV	41 (46.6)	19 (55.9)	
Max body temperature (Celsius degree)			≤ 0.001
Median (IQR)	37.0 (36–38)	38.0 (37.6–39.0)	
Range	36.0–42.0	36.0–40.0	
White blood cells count (× 10^3^/mmc)			≤ 0.001
Median (IQR)	10.9 (8.6–14.0)	14.3 (11.1–20.2)	
Range	1.1–29.0	3.3–53.0	
C-reactive protein (mg/dL)			≤ 0.001
Median (IQR)	4.0 (0.7–10.2)	14.6 (8.4–26.2)	
Range	0.1–52.0	0.2–47.5	
Serum creatinine (mg/dL)			0.3
Median (IQR)	1.4 (1.1–2.0)	1.5 (1.2–2.3)	
Range	0.6–9.7	0.2–5.9	
Perirenal fat stranding [No. (%)]	65 (73.9)	28 (82.4)	0.3
ROI area (mm^2^)			0.7
Median (IQR)	180.3 (128.6–262.2)	194.9 (134.4–267.3)	
Range	66.2–1451.4	61.4–631.4	
ROI perimeter (mm)			0.9
Median (IQR)	50.6 (44.3–62.5)	52.7 (41.2–60.4)	
Range	29.2–135.1	28.6–89.5	
Hounsfield unit value			≤ 0.001
Median (IQR)	2.7 (-0.1–7.6)	12.4 (7.3–16.9)	
Range	− 7.2 to 52.4	− 2.6 to 69.5	
Variability of Hounsfield unit			0.5
Median (IQR)	13.8 (12.2–15.4)	14.3 (12.2–16.8)	
Range	8.3–24.4	9.6–28.3	

BMI, body mass index; CCI, Charlson Comorbidity Index; ROI, region of interest.

*p value according to the Mann–Whitney test for continuous data and the Chi Square Test for categorical variables, as indicated.

**Figure 2:**
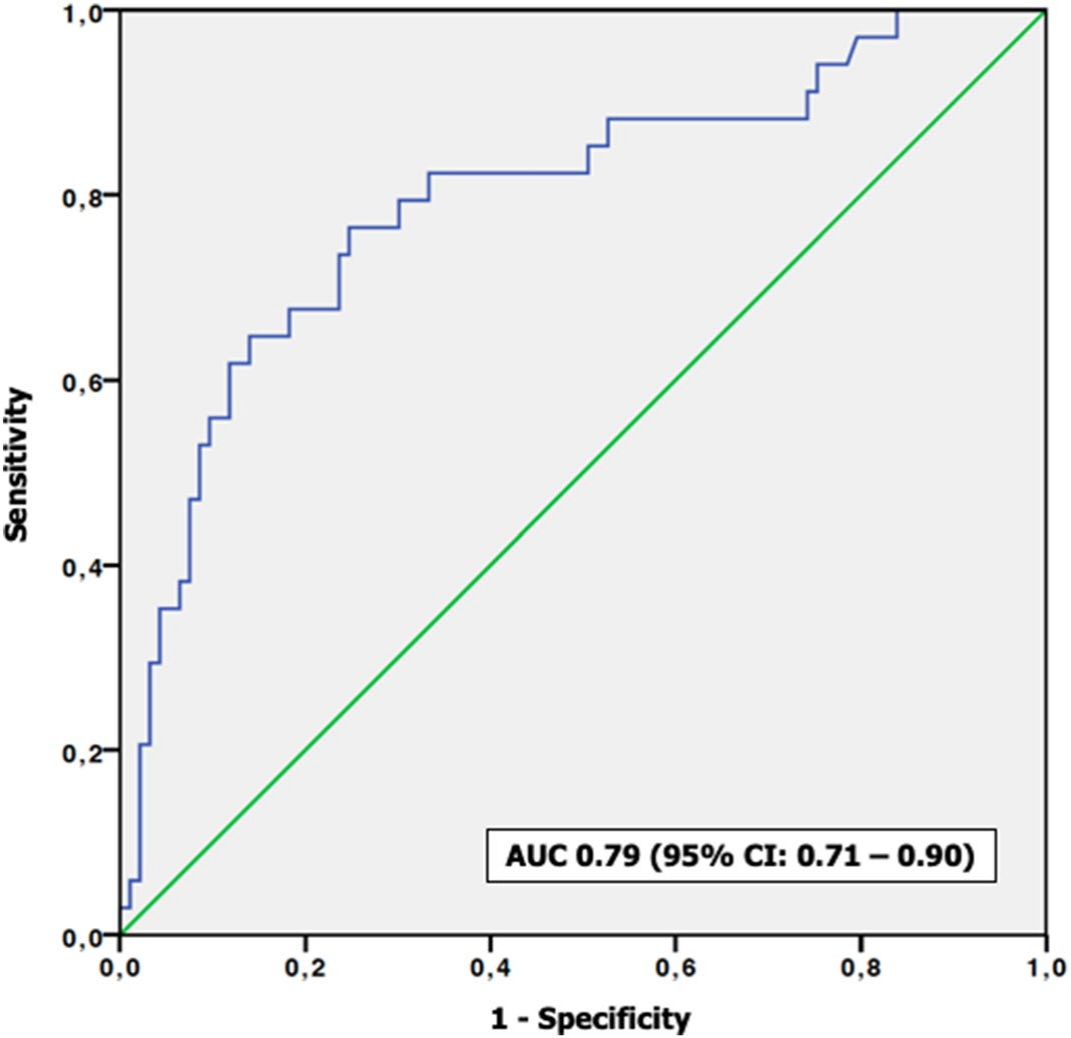
ROC analysis demonstrating the sensitivity and specificity of Hounsfield units (HU) in detecting sepsis after surgery.

ROC analysis showed that HU had a good ability to predict sepsis (AUC 0.79; 95%CI: 0.71–0.90) (Fig. 2). A HU cut-off value of 7.3 could diagnose the presence of sepsis with 76.5% sensitivity and 74.3% specificity.

At multivariable logistic regression analysis, CCI (OR 2.8; *p* = 0.01), WBC ≥ 15 × 10^3^/mmc (OR 2.8; *p* ≤ 0.001) and HU ≥ 7.3 (OR 7.35; *p* ≤ 0.001) were independently associated with sepsis (Table 5).

**Table 5:** Logistic regression models predicting sepsis in the whole cohort (OR; *p* value [95%CI]).

UVA model	MVA model			
	OR, *p* value	95% CI	OR, *p* value	95% CI
Age	1.12, 0.31	0.97–1.12		
BMI	1.01, 0.83	0.91–1.16		
Female gender	4.01, ≤ 0.01	1.70–9.23		
CCI ≥ 1	2.69, 0.01	1.11–6.34	2.81, 0.01	1.12–9.49
Aetiology				
Stone	Ref	Ref		
Urothelial tumours	1.10, 0.82	0.36–3.48		
Other	2.23, 0.31	0.42–9.89		
Grade III–IV	1.57, 0.32	0.71–3.57		
Vs. grade II hydronephrosis				
Perirenal fat stranding	1.50, 0.48	0.51–4.29		
Max body temperature	2.21, ≤ 0.001	1.41–4.12		
WBC ≥ 15 × 103/mmc	2.82, 0.01	1.11–6.41	2.87, ≤ 0.001	1.33–4.67
C-reactive protein	1.13, ≤ 0.001	1.06–1.43		
Serum creatinine	1.09, 0.72	0.81–1.38		
HU ≥ 7.3	9.37, ≤ 0.001	3.71–15.40	7.35, ≤ 0.001	2.56–16.78

UVA, univariate model; MVA, multivariate model; BMI, body mass index; CCI, Charlson Comorbidity Inex; WBC, white blood cells; HU, hounsfield unit.

## Discussion

We sought to assess the prevalence of PYO in a relatively large cohort of patients with obstructive uropathy and to analyse the potential role of HU of the hydronephrotic collecting system in predicting PYO presence and sepsis development after urinary drainage. Of clinical relevance, we found that approximately 40% of patients had clinical criteria suggestive for PYO in the real-life setting. In this context, PYO emerged to be associated with a greater CCI score, female gender and higher WBC and CRP values, thus suggesting a higher inflammatory burden. Patients with PYO had higher HU levels than those with HYDRO. HU determination showed a good predictive ability in differentiating PYO from HYDRO. We confirmed that PYO was associated with a higher risk of developing SIRS and sepsis^1,17^, therefore we tested the association between HU and sepsis. In this context we found that HU values were higher in patients with sepsis and that HU measurement (cut-off 7.3) could be used as a potential predictor for sepsis in patients with obstructive uropathy in the real-life setting. Preoperative CT-based HU measurement is a simple and cost-effective investigation that could be easily integrated in the diagnostic work-up of patients with obstructive uropathy.

Our interest was motivated by the lack of reliable clinical predictors of PYO in patients with obstructive hydronephrosis in the clinical practice. Indeed, nonspecific malaise or symptoms may be the only manifestations described in some cases^17,18^. Valid predictors of PYO, such as HU measurement, may help to prompt diagnosis and management of this potentially life-threatening condition.

Previous studies evaluated factors associated with PYO in patients with urinary stones. Patodia et al.^5^, analysed a cohort of 91 patients with PYO and 410 individuals without PYO treated at a single center for urinary stone disease and showed that risk factors for pyonephrosis were delayed presentation, large stone size, severe hydronephrosis and poor renal function. Our results corroborate these findings. We found that patients with PYO had higher degree of hydronephrosis than those -PYO. The severity of hydronephrosis emerged to be an independent predictor of PYO. Specifically, patients with grade III-IV hydronephrosis had threefold higher risk of PYO development than those with grade II dilation.

Obstruction and infection are two leading etiological mechanisms of PYO^1^; therefore any risk factor for urinary tract infections (UTI) could also promote pyonephrosis. For example it is known that UTI are more common in women than men^1,19^ and in individuals with higher numbers of comorbid conditions such as diabetes mellitus, hypertension and tumors^20,21^. We also found that patients with PYO had a higher CCI score, which indicates a higher comorbidity burden and were more frequently of female gender than HYDRO individuals.

HU measurement is routinely performed by physicians to evaluate the hardness of kidney stones^7,8^, plan stone treatment^11,12^ and to define renal masses^13,14^. Additionally, HU are used to characterize intra-abdominal fluid collections^22,23^. Gnannt et al. revealed that HU values, along with clinical and laboratory parameters, were useful for differentiating infected vs. non-infected abdominal fluids^22^. Similarly, CT attenuation values were found to be able to discriminate between exudates and transudates^23^.

In a recent study, Basmaci and Sefik^15^ analysed data from 31 patients treated with nephrostomy insertion for obstructive urinary tract infection and 22 individuals who underwent percutaneous nephrolithotomy for obstructive stones. All patients had renal pelvis urine collected at the time of kidney access. Authors found that individuals with positive urine culture had lower HU of the fluid in the dilated renal collecting system than those with negative culture. The best cut-off to predict a positive renal pelvis urine culture was zero^15^.

On the contrary, Yuruk et al.^6^ evaluated 105 patients with obstructive hydronephrosis treated with nephrostomy tube placement. Of 105, 47 (44.8%) individuals had clinical criteria suggestive for PYO. Authors reported that HU of the fluid in the dilated renal collecting system was higher in patients with PYO than HYDRO and that the HU cut-off value of 9.21 could identify PYO with 65.9% sensitivity and 87.9% specificity^6^. Our results are in line with these findings. We found that patients with PYO had higher HU values of the fluid in the dilated collecting system than those with HYDRO. The ROC curve analysis revealed that the HU value of 6.3 could diagnose the presence of PYO with 71.6% sensitivity and 71.5% specificity. Additionally, at multivariable logistic regression analysis HU ≥ 6.3 emerged to be an independent predictor for PYO after accounting for standard clinical and laboratory parameters.

From a preclinical standpoint, the rationale behind the use of HU values for differentiating PYO from HYDRO relies on the fact that the pyonephrotic fluid is composed of urine, infected material, cellular particles and microorganisms, all of which are able to increase the attenuation on a CT scan^17,24^. This may also explain the reason why having a positive renal pelvis urine culture, and not specifically pyuria itself, was found to be associate with low HU values^15^. In patients with renal stone, urine culture might be positive due to the presence of bacteria on the stone surface and not for the mechanical obstruction.

To the best of our current knowledge, this study is the first to show that HU of the fluid in the dilated collecting system are associated with a higher risk of septic complications in patients with obstructive uropathy. CT attenuation values of bladder and renal pelvis urine have been previously used to predict the culture positivity in patients with stone disease^15,16^ but the direct association between HU values and infectious complications after urinary drainage in the emergency setting has never been investigated in current literature. We found that patients who developed sepsis had higher preoperative HU values than those who did not experience infectious complications. Of note, we also showed that HU values were positively associated with the SOFA score, which is already known to be a prognostic factor of sepsis^25^. Interestingly enough, a HU cut-off value of 7.3 could diagnose the presence of sepsis with 76.5% sensitivity and 74.3% specificity. Moreover, patients with HU ≥ 7.3 had 8-times higher risk of sepsis, after accounting for clinical and laboratory parameters. From a speculative standpoint, higher HU values could be considered as a marker of a more severe PYO status which leads to a higher risk of sepsis.

Of clinical importance, HU value of the collecting system could be considered a reliable predictor of PYO and sepsis in patients with obstructive uropathy and could be used to prompt diagnosis and management of a severe condition that can lead to loss of kidney function and life-threatening complications.

The importance of our study as compared to previous reports is due to several aspects. First, we comprehensively analysed a relatively large cohort of patients with obstructive uropathy. As a matter of fact, we consistently assessed subjects via thorough clinical, laboratory and radiological investigations with the same methodological setting. On the contrary, other authors have not reported inflammatory markers or clinical parameters suggestive for UTI^6^, which limits the validity of their results. Second, HU measurements were performed in the NCCT phase in our study. Conversely, other Authors have analysed contrast-CT phases^6^, even though it is well known that the HU determination of the renal pelvis changes with contrast even at the early stage of parenchyma enhancement. Due to the use of NCCT scans our study appears to be more reproducible and our results may be more generalizable. Finally, we performed the first study with the specific aim of investigating the association between HU values and septic complications in patients with obstructive uropathy. Given the high risk of sepsis in this group^1^, finding reliable and easy-to-obtain predictors of infectious complications is a major clinical need. Patients at higher risk of developing sepsis might be managed with more intense monitoring of vital signs and early broad-spectrum antibiotics than those with lower risk that could be treated with less intensive care. As a whole, considering the potential life-threatening complications of obstructive uropathy, we recommend prompt drainage in any high-risk patient with ureteral obstruction.

Our study is not devoid of limitations. First, the results derive from a retrospective analysis of data prospectively collected, thus deserving external validation with an independent, larger and more diverse sample. Second, the decision to place a nephrostomy tube or a DJ was based on physician/patient preference and individuals’ clinical factors. We did not find any difference in preoperative and postoperative parameters according to the type of urinary drainage (data not shown) and previous reports showed no difference in septic complications after DJ or nephrostomy tube insertion in the setting of acute ureteral obstruction^26^. However, we cannot exclude that the difference in the urine collection method might have an impact on infectious outcomes in this cohort of patients. A prospective randomized controlled trial would be the ideal study design to evaluate differences in infectious complications after DJ or nephrostomy tube insertion for obstructive hydronephrosis. Third, as for common clinical practice, the diagnosis of pyuria was done by the direct visual assessment of the treating urologist without confirmatory lab testing. This could have biased the diagnosis of pyonephrosis in selected cases (i.e. prolonged urinary obstruction). Fourth, we lacked data on the group of patients with mild-moderate hydronephrosis that did not undergo surgery but was managed with conservative treatment. Lastly, despite HU evaluations being performed by two experienced radiologists in the slice with the maximal collecting system surface area paying great attention in order not to include adjacent renal parenchyma or stones into the measurement area, most of patients had grade II hydronephrosis, thus potentially leading to incorrect measurements. Moreover, since patients had different severity of hydronephrosis the size of the ROI was not standardized, thus potentially leading to some degree of variability in HU values.

In conclusion, the results of this cross-sectional, real-life study revealed that one out of three patients with obstructive uropathy showed clinical criteria suggestive for PYO. Patients with PYO had higher HU levels of the dilated collecting system than those with HYDRO. Higher degree of hydronephrosis, WBC count and HU values emerged to be independently associated with PYO status. This finding is relevant given the clinical importance of PYO, especially in the light of possible loss of kidney function and development of septic complications, which prompt early application of necessary countermeasures in the clinical practice. In this context we found that HU values have a good predictive ability for septic complications after urinary drainage. Patient’s comorbidity burden, WBC count and HU values emerged to be independent predictors of sepsis in patients with obstructive uropathy.

## Methods

### Patient population

We conducted a retrospective study at the Foundation IRCCS Ca’ Granda – Ospedale Maggiore Policlinico, in Milan, an academic tertiary referral center. We reviewed all data regarding patients that were consecutively admitted to our Emergency Department (ED) from September 2014 to June 2019 and underwent a urological evaluation (any reason). Analyzing the ED discharge records, patients were screened according to the diagnosis at discharge based on International Classification of Diseases, Ninth Revision, Clinical Modification (ICD‐9‐CM) codes^27^. We focused on urological and genito-urinary infections codes potentially associated with obstructive uropathy (Supplementary Table 3). For the specific purpose of this study we only included patients who underwent a CT scan at our institution and were treated with nephrostomy tube or ureteral catheter placement for obstructive uropathy.

All patients were assessed with a thorough medical history including age and comorbidities. Comorbidities were scored with the Charlson Comorbidity Index^28^. For the specific purpose of the analysis, CCI was categorised as 0 or ≥ 1. BMI, defined as weight in kilograms by height in square meters, was calculated for each patient.

Complete blood count and differential, platelet count, electrolytes, CRP, liver enzymes, serum protein, serum bilirubin and serum creatinine were measured in all patients.

According to our institutional policy, all patients had a CT scan before surgery for urinary decompression.

### Surgical technique

The treating urologist decided to place a nephrostomy tube or a ureteral catheter based on preference or patient factors. Parenteral broad-spectrum antibiotic prophylaxis was administered in all patients if not started in the ED before surgery. The nephrostomy tube was placed with the patient in supine position under ultrasound and X-ray guidance. After needle insertion in the collecting system, renal pelvis urines were collected for culture.

For ureteral catheter placement, a cystourethroscopy was initially performed and a hydrophilic guidewire was positioned into the renal pelvis under fluoroscopy guidance. A ureteral catheter was placed over the guidewire above the site of obstruction and urine was collected for culture. After performing a low-pressure retrograde pyelography to clearly identify the anatomy of the collecting system, a double J catheter (DJ) was positioned under fluoroscopic guidance. In cases where the guidewire or the ureteral catheter could not overcome the obstruction the procedure was promptly modified to nephrostomy tube placement.

Pyonephrosis was confirmed upon the observation of pyuria (cloudy/milky urine) following the insertion of the needle or the ureteral catheter^29^.

Blood cultures were collected in case of fever (max body temperature ≥ 38 °C) and/or chills before or after surgery. Postoperative sepsis was clinically defined as an acute increase in ≥ 2 Sequential Organ Failure Assessment (SOFA) points and documented blood or urine cultures^30^.

### Imaging technique

A dual source dual energy CT scanner (Siemens Somatom Definition Flash) was used for all cases. Unprocessed data acquired on axial plane with a slice thickness of 0.6 mm or 1.2 mm were processed and 3 mm slice axial images were obtained from the non-contrast CT phase (NCCT).

**Figure 3:**
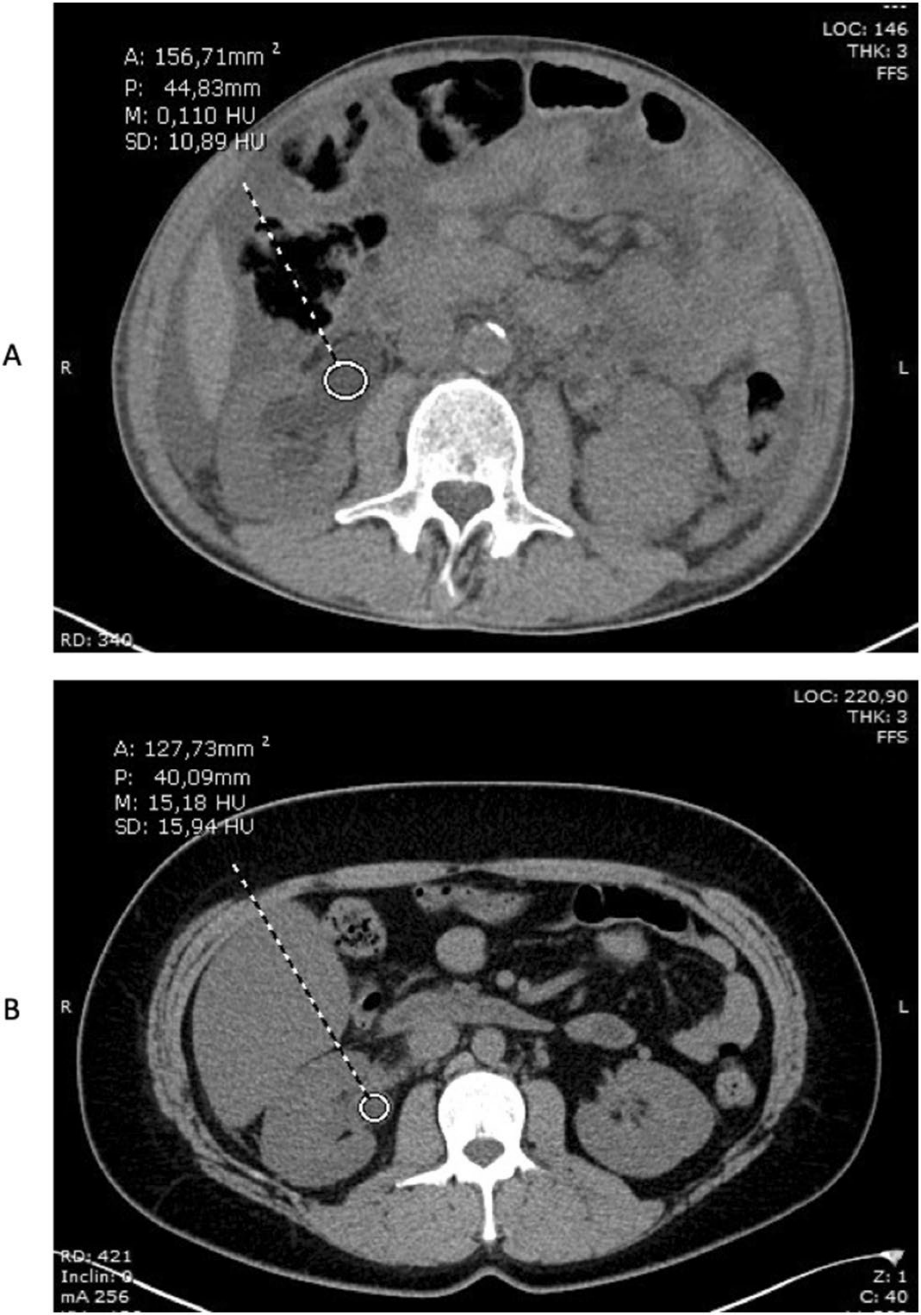
Hounsfield units measurement with an elliptical region of interest in the hydronephrotic region of the effected kidney. A = patient with obstructive hydronephrosis; B = patient with obstructive pyonephrosis. Legend: A = area; P = perimeter; M = mean; SD = standard deviation.

Two experienced Radiologists, blinded to each other, reviewed all CT images with PACS software in the absence of any information regarding the clinical and laboratory findings of the patients. Cases of interobserver disagreements in terms of grade of hydronephrosis and rate of perirenal fat stranding were resolved by a third party (G.C). Hydronephrosis was categorized according to the classification proposed by the Society for Fetal Urology^31^. An elliptical ROI was used for quantitative measurement of the HU values of hydronephrotic region in the slice with the maximal collecting system surface area of the effected kidney in soft tissue window (Fig. 3). HU measurement was performed in the NCCT phase because enhancement can change the attenuation values. ROI perimeter and elliptical area were also recorded. The physicians were very careful in order not to include adjacent renal parenchyma or stones into the measurement area. CT-based parameters were analyzed according to the mean of these values measured by the two radiologists.

Exclusion criteria were: preoperative CT scan not performed at our Institution, haematological or other diseases that could have altered blood tests, patients with grade I hydronephrosis and the presence of indwelling ureteral catheter before surgery (Supplementary Figure 2).

A convenient sample of 122 consecutive individuals evaluated at a single academic centre and treated between September 2014 and June 2019 was consider for final analysis.

Data collection followed the principles outlined in the Declaration of Helsinki. All patients signed an informed consent agreeing to share their own anonymous information for future studies. The study was approved by the Foundation IRCCS Ca’ Granda – Ospedale Maggiore Policlinico Ethical Committee (Prot. 25,508).

### Statistical analyses

Distribution of data was tested with the Shapiro–Wilk test. Descriptive statistics of categorical variables focused on frequencies and proportions. Medians and Interquartile Ranges were reported for continuously coded variables.

Interobservers agreement for radiological parameters was evaluated with the Intraclass Correlation Coefficient and the Wilcoxon Signed Rank Test. The Mann–Whitney test and Chi Square test were used to assess potential differences in terms of clinical, laboratory and radiographic parameters in patients with HYDRO vs. PYO. Receiver Operating Characteristic curves were generated to find HU value cut-offs (defined as Youden J Index) to predict PYO status. Binary logistic regression analyses tested the association between clinical predictors (e.g. gender, body temperature, WBC count, grade of hydronephrosis and HU values) and the presence of PYO.

Similarly, descriptive statistics and ROC curves evaluated the association between HU values and sepsis. Logistic regression analyses were used to test the association between clinical predictors (e.g. CCI, WBC, and HU values) postoperative sepsis, clinically when infection was suspected. Statistical tests were performed using SPSS v.26 (IBM Corp., Armonk, NY, USA). All tests were two sided, with a significance level set at 0.05.

## Supplementary information

Supplementary Tables.

Supplementary Figure 1.

Supplementary Figure 2.

## Data Availability

All relevant data are within the paper and its Supporting Information files.
