# Supplementary material for: Hounsfield unit attenuation value can differentiate pyonephrosis from hydronephrosis and predict septic complications in patients with obstructive uropathy
Source: Sci Rep. 2020 Oct 29;10:18546. doi: 10.1038/s41598-020-75672-8 (PMC7596071; doi:10.1038/s41598-020-75672-8)
Supplement: Supplementary file 1 — Supplementary Tables. [file 41598_2020_75672_MOESM1_ESM.doc]

**Hounsfield unit attenuation value can differentiate pyonephrosis from hydronephrosis and predict septic complications in patients with obstructive uropathy**

Luca Boeri^1*^, Irene Fulgheri^2^, Franco Palmisano^1^, Elena Lievore^1^, Vito Lorusso^1^, Francesco Ripa^1^, Mario D’Amico^3^, Matteo Giulio Spinelli^1^, Andrea Salonia**^4^**, Gianpaolo Carrafiello^3^, Emanuele Montanari^1^

**Supplementary Table 1: Descriptive characteristics of stones causing obstructive uropathy in 97 patients**

Left/Right side 46/51

Stone size (mm)

Median (IQR) 7 (4-9)

HU of stone

Median (IQR) 850 (400-1500)

Stone location [No. (%)]

Proximal 41 (42.3)

Mid 23 (23.7)

Distal 33 (34.0)

Keys: HU = Hounsfield Unit

**Supplementary Table 2: Urine culture results in the whole cohort (No. = 122)**

**Hydronephrosis Pyonephrosis**

Negative 65 (85.5%) 6 (13.0%)

E.coli 9 (11.8%) 29 (63.1%)

Klebsiella pneumonia 0 (0.0%) 3 (6.5%)

Enterococcus faecalis 1 (1.3%) 5 (10.9%)

Proteus Mirabilis 0 (0.0%) 1 (2.2%)

Pseudomonas Aeruginosa 1 (1.3%) 2 (4.3%)

**Supplementary Table 3. The International Classification of Diseases, Ninth Revision, Clinical Modification (ICD-9-CM)**

Urological diagnosis code

599.60 Urinary Obstruction, Unspecified

599.69 Urinary Obstruction, Not Elsewhere Classified

599.89 Other specified disorders of urinary tract

591 Hydronephrosis

592.0 Kidney Stone

592.1 Ureteral Calculus

592.9 Urinary calculus, unspecified

593.3 Stricture or kinking of ureter

593.4 Other ureteric obstruction

594.9 Calculus of lower urinary tract, unspecified

788.0 Renal colic

Genito-urinary infections

590.10 Pyelonephritis, Acute w/o Necrosis

590.80 Pyelonephritis, unspecified

590.81 Pyelitis or pyelonephritis in diseases classified elsewhere

599.0 Urinary Tract Infection
