# Supplementary figures and images for: Hounsfield unit attenuation value can differentiate pyonephrosis from hydronephrosis and predict septic complications in patients with obstructive uropathy
Source: Sci Rep. 2020 Oct 29;10:18546. doi: 10.1038/s41598-020-75672-8 (PMC7596071; doi:10.1038/s41598-020-75672-8)

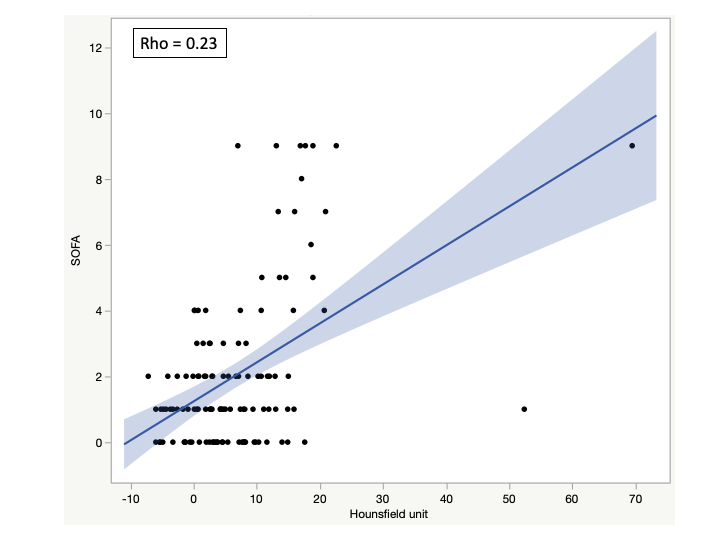

Supplement: Supplementary file 2 — Supplementary Figure 1. [file 41598_2020_75672_MOESM2_ESM.tiff]

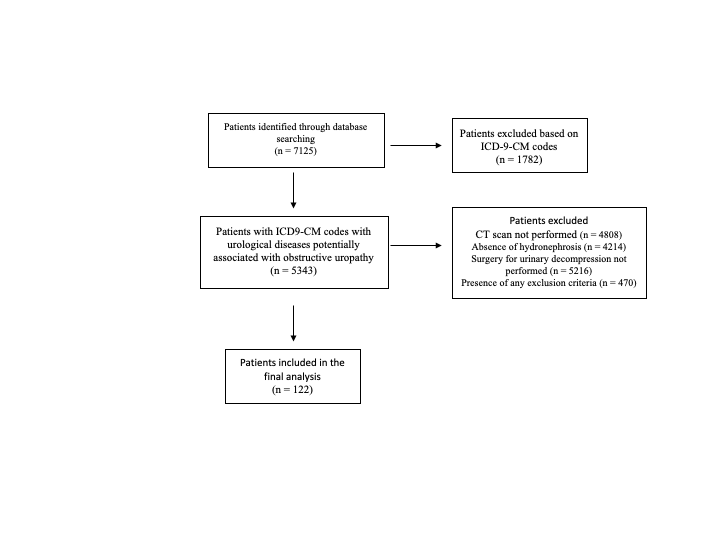

Supplement: Supplementary file 3 — Supplementary Figure 2. [file 41598_2020_75672_MOESM3_ESM.tiff]
